# Sample-minimizing co-flow cell for time-resolved pump–probe X-ray solution scattering

**DOI:** 10.1107/S1600577522012127

**Published:** 2023-02-01

**Authors:** Irina Kosheleva, Robert Henning, Insik Kim, Seong Ok Kim, Michael Kusel, Vukica Srajer

**Affiliations:** a BioCARS, Center for Advanced Radiation Sources, The University of Chicago, 9700 South Cass Ave, Bld 434B, Lemont, IL 60439, USA; b Department of Chemistry, Korea Advanced Institute of Science and Technology, E6-6 #513, 291 Daehak-ro, Daejeon, Yuseong-gu 34141, Republic of Korea; c Kusel Design, 12 Coghlan Street, Niddrie, Wurundjeri Country 3042, Australia; University of Tokyo, Japan

**Keywords:** sheath co-flow cell, time-resolved pump–probe X-ray solution scattering, sample minimization

## Abstract

A fundamental problem in biological sciences is understanding how macromolecular machines work and how the structural changes of a molecule are connected to its function. Time-resolved techniques are vital in this regard and essential for understanding the structural dynamics of biomolecules. Time-resolved small- and wide-angle X-ray solution scattering has the capability to provide a multitude of information about the kinetics and global structural changes of molecules under their physiological conditions. However, standard protocols for such time-resolved measurements often require significant amounts of sample, which frequently render time-resolved measurements impossible. A cytometry-type sheath co-flow cell, developed at the BioCARS 14-ID beamline at the Advanced Photon Source, USA, allows time-resolved pump–probe X-ray solution scattering measurements to be conducted with sample consumption reduced by more than ten times compared with standard sample cells and protocols. The comparative capabilities of the standard and co-flow experimental setups were demonstrated by studying time-resolved signals in photoactive yellow protein.

## Abstract 2

For irreversible or slow reactions studies by time-resolved pump–probe X-ray solution scattering, the standard sample cell and protocol for data collection at the BioCARS 14-ID beamline potentially require up to hundreds of milligrams of sample. A sheath co-flow cell, described here, allows such solution scattering measurements to be conducted with the sample consumption reduced by more than ten times compared with standard protocols at this beamline.

## 1. Introduction

Biomolecules undergo structural changes in response to modulations in their environment, for example temperature, concentration of protons (pH) or ions of metals, ligand binding, light absorption, electric field and other stimuli. Recording the molecular structural response as a function of time after such modulations allows the molecular structural intermediates during the reaction pathway, their time courses and free-energy landscape to be determined (Thompson *et al.*, 2019 ▸; Ihee *et al.*, 2005 ▸; Schmidt *et al.*, 2013 ▸; Akiyama *et al.*, 2002 ▸; Andersson *et al.*, 2009 ▸; Kim *et al.*, 2012*a* ▸). It is favourable to know the molecular structural response in most detail. Time-resolved crystallography provides atomic resolution structures of such molecular intermediates (Brändén & Neutze, 2021 ▸; Srajer & Schmidt, 2017 ▸) but large functional structural changes may not be accessible due to crystal packing forces. In addition, the structural dynamics in the crystal may differ from the dynamics in the solution environment (Cho *et al.*, 2016 ▸). X-ray solution scattering has the advantage of probing a wider range of structural dynamics in molecules. However, due to the random orientation of molecules in solution, their interference pattern is averaged azimuthally, so only radial structural information is preserved (Putnam *et al.*, 2007 ▸; Koch *et al.*, 2003 ▸). Macromolecules operate over large structural and temporal scales, from electron transfer processes on the sub-picosecond time scale to large conformational changes that may continue for hundreds of seconds and longer. Examples include protein folding and conformational dynamics (Wee *et al.*, 2012 ▸; Srajer & Royer, 2008 ▸; Olmos *et al.*, 2018 ▸; Hsu *et al.*, 2021 ▸), ligand and RNA/DNA binding (Wee *et al.*, 2012 ▸; Tokuda *et al.*, 2018 ▸), signal transduction (Cho *et al.*, 2016 ▸; Takala *et al.*, 2014 ▸), electron transfer mechanisms (Rimmerman *et al.*, 2018 ▸; Cammarata *et al.*, 2008 ▸) *etc*. The most common way to study molecular structural dynamics in solution is by mixing components or changing the pH or ionic strength of the solvent *etc.* and following the reaction by using optical or X-ray scattering methods. If molecules of a solute or a solvent are photosensitive by nature or by design, the reaction can be initiated by a laser pulse, and then probed optically or by X-rays. This time-resolved technique is referred to as pump–probe X-ray solution scattering.

Advances in improving the brilliance of X-ray sources, combined with fast detectors and technical advances in X-ray focusing, made possible the use of microfluidic mixing devices for studying molecular dynamics by time-resolved small-angle X-ray scattering (SAXS). Significant efforts have been made to improve laminar and turbulent mixers (Köster *et al.*, 2008 ▸; Inguva *et al.*, 2018 ▸; Kathuria *et al.*, 2013 ▸; Mizukami & Roder, 2022 ▸; Ghazal *et al.*, 2016 ▸, Park *et al.*, 2006 ▸; Plumridge *et al.*, 2018 ▸). The time resolution in both turbulent and laminar devices is limited by the diffusion time across the channel, and time series are collected by scanning the mixing cell across the X-ray beam. Advances in the design of turbulent mixers allowed the time resolution to be increased in mixing experiments to ∼30–50 µs (Graceffa *et al.*, 2013 ▸; Akiyama *et al.*, 2002 ▸; Matsumoto *et al.*, 2007 ▸). Turbulent mixing demands high flow rates to achieve breaking the liquid into small eddies (Graceffa *et al.*, 2013 ▸) for fast mixing. As a result, turbulent mixing requires very high sample consumption. Therefore, most mixing experiments performed using this technique use commercially available samples. In laminar mixers, sample consumption is small compared with turbulent mixing. Typically, in laminar mixers, the central sample stream is focused hydro­dynamically by a series of buffer streams. Laminar mixers require very narrow sample streams, typically only several micrometres in diameter, to decrease the diffusion time. Several types of laminar mixers have been developed by different groups (Brody *et al.*, 1996 ▸; Pollack *et al.*, 2001 ▸; Köster *et al.*, 2008 ▸; Park *et al.*, 2006 ▸; Plumridge *et al.*, 2018 ▸). So far, sub-millisecond time resolution in laminar mixers has been achieved (Park *et al.*, 2006 ▸), but the millisecond range is typical. Microfluidic mixers, turbulent or laminar, remain custom-built state-of-the-art devices, micro-machined, etched or printed (Ghazal *et al.*, 2016 ▸; Lu *et al.*, 2016 ▸, Plumridge *et al.*, 2018 ▸).

The reaction photo-initiation method employed in pump–probe time-resolved X-ray solution scattering experiments is distinctly different from the reaction initiation by mechanical mixing in mixing devices, either turbulent or laminar. Subsequently, the design requirements for those two types of apparatuses are also incisively different (Huyke *et al.*, 2020 ▸). In this contribution, we discuss solely the pump–probe time-resolved X-ray solution scattering (TRXSS) technique. In TRXSS, a reaction in a molecule is triggered by an external short stimulus pump, often a laser pulse, and is probed by a delayed short X-ray pulse probe. Then the sample volume is refreshed and the process is repeated. We refer to the frequency at which the pump–probe is repeated as the repetition rate of the experiment. A complete time-resolved pump–probe experiment typically involves collecting scattering data at a number of time delays between pump and probe pulses. The length of the X-ray probe or the length of the laser pump, whichever is longer (typically the X-ray probe pulse is longer), defines the time resolution. At dedicated time-resolved pump–probe beamlines, a difference technique is used to measure scattering before and after reaction initiation with great accuracy and with a time resolution of 100 ps (at third- and fourth generation synchrotron sources) or tens of femtoseconds (at X-ray free-electron lasers) (Kim *et al.*, 2011 ▸, 2012*a* ▸; Cammarata *et al.*, 2008 ▸; Lee *et al.*, 2021 ▸; Levantino *et al.*, 2015*a* ▸).

To achieve the best time resolution at the BioCARS 14-ID beamline at the Advanced Photon Source (APS), USA, we use a picosecond or nanosecond laser pulse as a pump and a single polychromatic X-ray pulse of 100 ps duration as a probe. This permits a reaction to be probed in the time window of 100 ps to a few hundreds of nanoseconds. However, even with the high polychromatic X-ray flux at 14-ID, a single 100 ps X-ray pulse is not sufficient to achieve the necessary signal-to-noise ratio and a number of pump–probe cycles have to be repeated and data accumulated. For a typical experiment, one needs to accumulate data from thousands of single X-ray pulses per image and hundreds of images to achieve the signal-to-noise required to see differences in SAXS scattering curves. The standard capillary cell diameter used in such experiments at 14-ID at 12 keV is ∼0.5–1 mm. For irreversible reactions or reactions with slow initial-state recovery such as enzymatic reactions, the sample has to be refreshed between pump–probe cycles. When experiments are conducted with a standard capillary flow cell, the sample consumption for the complete experiment can be as high as several grams of protein. This is an unsustainable amount for protein produced in a research lab. So far, most experiments for pump–probe TRXSS have been performed with X-ray radiation stable samples that undergo reversible reaction, such as photoactive yellow protein, cytochrome *c*, myoglobin *etc*. However, many biological reactions are irreversible or have very long relaxation times. To render those irreversible reaction studies possible, we have developed a sample-minimizing cytometry-style sheath co-flow device suitable for pump–probe TRXSS experiments.

## 2. Sheath co-flow cell description

In the co-flow cell, a sample solute is injected through a tube or a needle into a capillary filled with a solvent. The co-flow-type cell has been widely used in Taylor dispersion analysis, cytometry, cell sorting or alignment, fluorescence flow-induced dispersion analysis, time-resolved spectroscopy mixing experiments and other applications (Ghazal *et al.*, 2016 ▸; Buell & Jensen, 2021 ▸; Gang *et al.*, 2018 ▸; Wang *et al.*, 2014 ▸; Calvey *et al.*, 2019 ▸; Hamadani & Weiss, 2008 ▸; Huyke *et al.*, 2020 ▸). Co-flow mixing cell designs were also employed in time-resolved solution scattering mixing experiments (Pollack *et al.*, 2001 ▸; Park *et al.*, 2006 ▸, 2008 ▸; Plumridge *et al.*, 2018 ▸). In static X-ray solution scattering experiments, a co-flow device was used for decreasing radiation damage of the samples during static SAXS measurements (Kirby *et al.*, 2016 ▸).

At BioCARS, the goal of introducing the co-flow cell design was to minimize sample consumption in time-resolved pump–probe X-ray solution scattering measurements. The cell has to satisfy several requirements. The sample and the buffer flow in the cell must be laminar in the range of velocities required in a typical pump–probe experiment with repetition rates up to 1 kHz. In addition, it has to be compatible with anaerobic sample studies and deliver stable sample streams of a variety of sizes, which match the sizes of the X-ray and laser beams at the beamline. The sample cell has to be robust in handling, made from parts available commercially and be compatible with BioCARS laser configurations (collinear and orthogonal to the X-ray beam), X-ray path and visualization optics. In partnership with Küsel Design (Australia), we designed and produced such a robust flow cell, which was integrated into the BioCARS 14-ID beamline.

**Figure 1:**
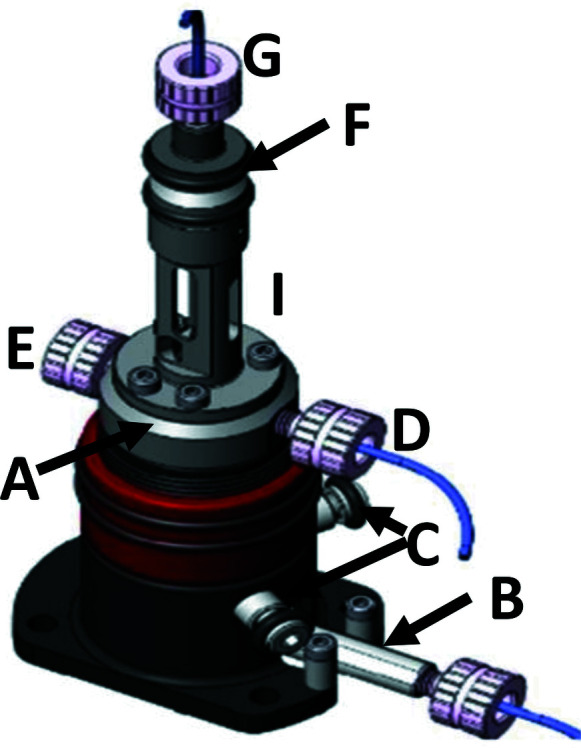
Drawing of the co-flow cell for time-resolved pump–probe X-ray solution scattering

A drawing of the co-flow cell is shown in Fig. 1 ▸. The device has a modular design. The main body of the sample consists of a quartz capillary with diameter of 0.6 mm and wall thickness of 10 µm. The capillary inlet is connected to the upper chamber (A). The chamber houses a stainless-steel needle with ∼200 µm orifice for sample delivery (B); the needle position has manual adjustment screws (C) which allow the centre of the needle to be aligned in the capillary. The upper chamber also connects to a buffer line (D) and a vent line (E). All connections accept standard 1/16-inch outer-diameter tubing fittings. The capillary outlet attaches to the bottom enclosure (F) and to a discard line (G). The connection of the sample cell capillary to the inlet and outlet enclosures are sealed with Viton O-rings. The whole assembly is mounted in a rigid stainless-steel enclosure (I) which has openings for access to pump laser beam, probe X-ray beam and viewing optics. The sample and the buffer are pumped in a controlled manner through the flow cell. An important feature of the design is that it constitutes a closed volume, essential in the experiments, which require an anaerobic sample environment (Rimmerman *et al.*, 2018 ▸; Kim *et al.*, 2011 ▸; Levantino *et al.*, 2015*b* ▸).

The flow dynamics of incompressible liquids in a co-flow capillary system are well understood (Lu *et al.*, 2016 ▸). When fluid is incompressible, has constant viscosity and is in a laminar regime, the liquid flows in parallel layers without interaction between them, and each layer flows with different velocity along the same direction. Neglecting the effects of gravity, one can obtain equations for the buffer and sample flow velocities (Landau & Lifshitz, 1987 ▸),

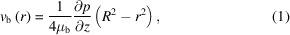




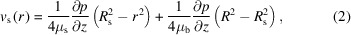

where *R* is the radius of the capillary, *R*_s_ is the radius of the sample core, μ_s_ is the sample viscosity, μ_b_ is the buffer viscosity, and ∂*p*/∂*z* is the pressure drop in the capillary. Then the ratio of buffer to sample flow rates (*Q*_b_ and *Q*_s_) follows the simple equation

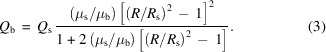

**Figure 2:**
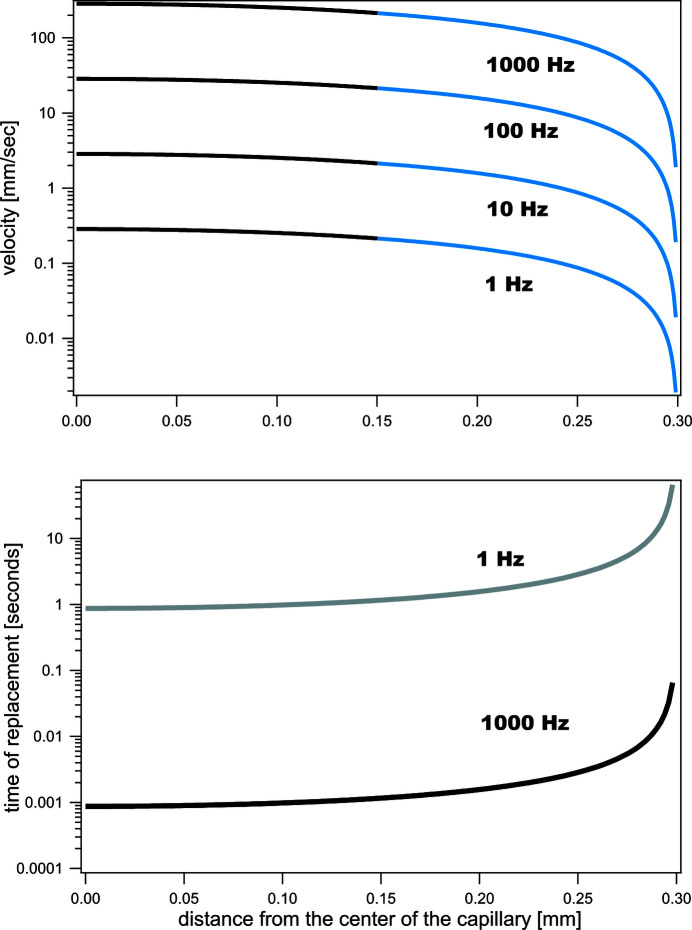
Velocity (top panel) and time of sample refreshment (bottom panel) as functions of distance from the centre of the capillary are shown for different repetition rates of the experiment. For the co-flow cell a buffer, velocities of which are shown in blue, occupies the outer portion of the capillary cell.

In time-resolved pump–probe experiments, the X-ray pulse has to probe the sample volume in which the reaction was initiated by the laser pulse. To ensure the most homogeneous laser pulse exposure of the sample volume probed by the X-ray pulse, the laser beam size is normally slightly larger than the X-ray beam size. Therefore, the average linear speed of the sample refreshment is defined by the laser spot size and the time between the pump–probe cycles (repetition rate of an experiment). As seen from equations (1) and (2), the linear sample speed and sample core diameter uniquely define the buffer linear speed. Fig. 2 ▸, top panel, shows calculated speeds for sample and buffer solutions that are needed for sample refreshment for typical repetition rates. Speeds are shown as a function of the distance from the centre of the capillary. The calculations were performed for the 0.6 mm capillary device and a target 0.3 mm sample core diameter, 0.25 mm laser beam size, and repetition rates ranging from 1 Hz to 1 kHz. In the calculations, viscosities of the sample and the buffer are assumed to be equal and gravity is neglected. If the gravitational force is parallel to the direction of the flow, differences in density of the sample solution and buffer solute cause small corrections to calculated values (Giorello *et al.*, 2020 ▸). In the case when the gravitational force is perpendicular to the direction of the flow, the result may be complicated. For small molecules, this may cause mixing in the vertical direction, as a function of distance from the sample injection.

For the time-resolved pump–probe measurements, it is important that the flow remains laminar and there is no intermixing between the layers. In this regard, an important characteristic of the device is the Reynolds number, Re. When Re is low, viscous forces dominate and liquid moves in a laminar manner. If Re is greater than ∼2 × 10^3^ (Squires & Quake, 2005 ▸) the flow becomes turbulent. For the velocities shown in Fig. 2 ▸, and assuming a kinematic viscosity of water at 20°C of ∼1 mm^2^ s^−1^, the co-flow device has an Re value below 200; therefore, the laminar condition is well satisfied. With low sample speeds, diffusion will play a larger contribution in the accuracy of time-resolved pump–probe measurements. The translational diffusion coefficient is typically 10^−5^ cm^2^ s^−1^ for small molecules and 10^−7^ cm^2^ s^−1^ for large macromolecules. For lower repetition rates, and therefore small sample linear speeds, the diffusion length for species with high diffusivity may become comparable with the sample core size, effectively causing sample dilution. This dilution does not constitute a problem if the sheath buffer is matching the protein buffer and if dilution does not change the solute–solvent and solute–solute interactions.

In a co-flow cell capillary, only the buffer is in contact with the capillary walls and only the buffer velocity approaches zero. Under such conditions, there is no capillary wall fouling and there is no sample radiation damage in the proximity of the capillary walls (Kirby *et al.*, 2016 ▸). Fig. 2 ▸, lower panel, shows the time required to replenish the liquid sample inside the capillary as a function of the distance from the capillary centre. The time was calculated for the 0.6 mm capillary and a laser beam size of 0.25 mm at repetition rates of 1 Hz and 1 kHz. In laminar flow devices, the sample refreshment time at the capillary wall approaches infinity so the sample is not refreshed between the pump–probe cycles. This portion of the sample experiences multiple pump–probe cycles during the data collection. Fig. 2 ▸ shows that as much as ∼15% of the total sample in the proximity of the capillary wall may not be properly refreshed. This portion of the sample experiences radiation damage and contributes to the low scattering vector region of the difference signal. In addition, if the relaxation time of the molecular reaction is longer than the repetition rate of the experiment, the sample will not be excited by the laser pump and therefore will be excluded from the reaction course. Both contributions will change the magnitude and the time course of the measured time-resolved structural changes. Although these have not caused issues for most experiments at BioCARS, the effects were observed in some cases. In the co-flow device, because the sample occupies only the central portion of the capillary, these effects are significantly minimized, or eliminated.

## 3. Data collection

To characterize the co-flow cell performance, we compared time-resolved scattering data for the photoactive yellow protein (PYP) collected with the co-flow cell and with a standard flow cell. PYP is a small photoreceptor protein. The photocycle reaction in PYP starts with absorption of blue light by a pCA chromophore molecule and isomerization of the chromophore from *trans* to *cis*. The structural signal then propagates through multiple intermediate states, which are well characterized spectroscopically and structurally (Ihee *et al.*, 2005 ▸; Kim *et al.*, 2012*a* ▸; Schotte *et al.*, 2012 ▸; Schmidt *et al.*, 2013 ▸; Meyer *et al.*, 1987 ▸; Ujj *et al.*, 1998 ▸). The sample in this study was 1.5 m*M* PYP solution in a buffer of 20 m*M* Tris, pH 7.0, 150 m*M* NaCl. PYP was prepared as previously described (Yang *et al.*, 2017 ▸). For the co-flow cell data collection, the matching 20 m*M* Tris, 150 m*M* NaCl buffer at pH 7.0 was used.

Time-resolved pump–probe X-ray solution scattering data were collected at BioCARS 14-ID-B beamline at the APS, in standard 24-bunch operating mode of the storage ring. The beamline has two pairs of Kirkpatrick–Baez mirror systems capable of focusing X-ray beam down to ∼15 µm × 20 µm (V × H) at the sample position, and timing shutters which can deliver on demand a single 100 ps X-ray pulse or a longer X-ray pulse sequence, with frequency of up to 1 kHz (Graber *et al.*, 2011 ▸). In standard time-resolved solution scattering configuration at 14-ID-B, polychromatic X-ray beam is used at peak energy 12 keV and bandpass of ∼2.5%. A single 100 ps polychromatic X-ray pulse delivers 5 × 10^9^ photons. Standard data collection requires 50–100 images per one time delay, with approximately 2000 single 100 ps X-ray pulses per image. For such data collection, sample consumption can be as high as 20–40 ml per one time delay when a standard diameter flow cell is used. Because the velocity of the sample replacement at the walls of the capillary approaches zero, it is not uncommon to expend even more sample to ensure proper sample replacement between pump–probe cycles.

**Figure 3:**
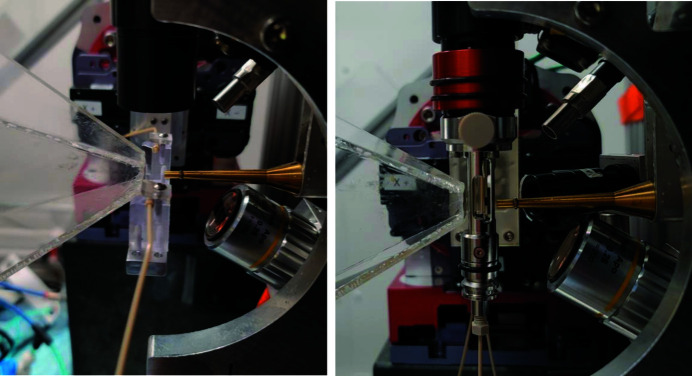
Sample environments for the standard flow cell (left panel) and co-flow cell (right panel).

The sample environments of the experiment in the standard flow cell and co-flow cell data collection modes are shown in Fig. 3 ▸. An Opotek nanosecond laser with 7 ns pulse duration tuned to 450 nm was used to initiate the reaction in the sample. The X-rays were focused to a spot size of 35 µm × 35 µm (FWHM). The scattering pattern was recorded using a Rayonix HS-340 detector in 2 × 2 binning mode (pixel size 88.6 µm). A cone purged with He gas was mounted on the face of the detector to minimize air scattering. Solution scattering was measured in the range of the scattering vector *q* = 4π sin(θ)/λ (where 2θ is the scattering angle and λ is the X-ray wavelength) from 0.02 Å^−1^ to 2.8 Å^−1^.

To enable direct comparison between the measurements, data collection in standard mode and co-flow mode were carried out at the same repetition rates of 20 Hz and 5 Hz. At 5 Hz we collected a time delay series consisting of longer pump–probe delays: −10 µs, −5 µs, 5 µs (standard mode only), 50 µs (co-flow mode only), 500 µs, 5 ms, 50 ms and 150 ms. The 5 Hz repetition rate (200 ms between pump–probe sequences) for these time delay series was dictated by the longest time delay in the series (150 ms). Given that we probed longer pump–probe time delays, we also used longer X-ray exposures as probes. Rather than a single 100 ps X-ray pulse, we used an X-ray probe consisting of 24 consecutive 100 ps X-ray pulses, with total duration of 3.6 µs. We refer to this probe as a 24-pulse X-ray exposure. At a faster 20 Hz repetition rate we collected shorter pump–probe time delays: −5 µs, −10 µs, 5 ns (co-flow mode only), 50 ns (standard mode only), 500 ns and 500 µs. The short time delays in this series required using a single 100 ps X-ray pulse as a probe (using several consecutive X-ray pulses as a probe was not possible in this case as pulses are spaced at 150 ns in the APS storage ring mode we used). We refer to this probe as a single-pulse X-ray exposure. A repetition rate of 20 Hz was determined by the maximum repetition rate of the Opotek nanosecond laser we used. We would like to emphasize here that, for both 5 Hz and 20 Hz data collections, one pump–probe cycle and therefore one X-ray exposure (as defined above for two repetition rates) is not sufficient for recording an image with acceptable signal-to-noise level. Pump–probe cycles have to be repeated and accumulated prior to an image readout. Details of how this is accomplished for standard and co-flow cells are described in Sections 3.1 and 3.2, respectively. Laser-OFF images were obtained at −5 µs and −10 µs time delays, meaning that first the X-ray image was collected and then the laser pulse arrived after a specified time delay. X-ray solution scattering is known to be very sensitive to temperature, with a significant temperature structural signal contribution at high scattering angles, reflecting changes in interatomic distances between the solvent molecules. We use laser-OFF negative time delays to keep capillary temperature conditions as close as possible to the laser-ON pump–probe measurements and to minimize possible experimental errors due to capillary local temperature instability. During initial data reduction, geometrical and polarization corrections were applied; then images were integrated radially to obtain 1D scattering curves and normalized on the isosbestic point of the solvent in the scattering vector *q* range 1.4–1.65 Å^−1^. For each laser-ON pump–probe scattering curve, the appropriate laser-OFF scattering curve was subtracted. This difference technique mitigates experimental errors caused by the slow changes in experimental conditions such as small temperature drifts, beam positions instabilities *etc*. The common time delays were used to scale data collected at different frequencies.

### 3.1. Standard data collection mode

In standard data collection mode, the nanosecond laser was focused to an elliptical spot size of 120 µm × 500 µm (FWHM), with power density of 1 mJ mm^−2^ and the shorter axis of the elliptical spot along the length of the capillary. Polychromatic X-ray beam was used in the direction perpendicular to the laser beam and the direction of the capillary cell. The laser and X-rays were aligned to overlap spatially at the sample position. In this setup we used a standard flow cell of 0.5 mm-diameter quartz capillary and a wall thickness 10 µm. The observed structural signal was maximized by collecting data at 0.15 mm from the top of the capillary. To minimize radiation damage of the sample and to ensure full sample recovery between the pump–probe cycles, the cell was translated through the X-ray beam with step size 0.25 mm at 5 Hz or 20 Hz repetition rates (see details below). After the capillary scan, 20 µl of sample in the capillary cell was refreshed, and the process was repeated until the desired quantity of the total X-ray pulses per image was achieved. Then the image was read by the detector. In total, the number of single 100 ps X-ray pulses per image equals the product of the number of single X-ray pulses in an X-ray exposure used for a pump–probe step, the number of pump–probe steps per capillary scan, and the number of capillary scans per image. For shorter time delays, we used a 20 Hz repetition rate, a single-pulse exposure per pump–probe step and 107 pump–probe steps per capillary scan. After the capillary scan, the sample was replenished. This procedure was repeated ten times before the image was finally read. In this case, the total number of single 100 ps X-ray pulses per image was therefore 1 single pulse per exposure × 107 pump–probe steps per capillary × 10 capillary scans. Therefore 1070 single 100 ps X-ray pulses were used per image, with a total of 10 × 20 µl = 200 µl of sample solution per image. For 5 Hz data collection, we used a 24-pulse exposure per pump–probe step (see description in Section 3) and also used 107 pump–probe steps per capillary scan. In this case the image was read after each scan. The total number of single 100 ps X-ray pulses per image was 24 single pulses per exposure × 107 pump–probe steps per capillary, and therefore 2568 single 100 ps X-ray pulses per image. In this case 20 µl of sample per image was used (see Table 1 ▸ for details). For each time delay, we collected and averaged 50 images at both repetition rates.

### 3.2. Sheath co-flow cell data collection mode

For co-flow data collection, given the vertical mount of the co-flow cell, the laser light was delivered in near-collinear geometry, at ∼15° with respect to the X-ray beam direction. The laser was focused into a 150 µm × 150 µm spot with laser power density of 1 mJ mm^−2^. The sample solution and the buffer were pumped through the capillary cell using a CP Dual Syringe (Cole-Parmer) which allows two liquids with different flow rates to be delivered simultaneously. The sample solution was injected through a needle in the centre of the co-flow cell while buffer was delivered through the buffer port as described above. To avoid sample flow instabilities, the solutions were degassed by bubbling nitro­gen through the liquids for 20 min prior to data collection. The co-flow cell was used in the vertical geometry so the gravity force was collinear with the directions of sample and buffer flows. The flow rate of the sample delivery pump was chosen so that fresh sample was delivered at each pump–probe cycle, and the targeted sample core was 300 µm in diameter. The buffer flow rate was calculated using equation (3). The sample core size was measured by scanning the capillary horizontally across the beam and measuring the SAXS signal, and then the capillary was centred. As for the standard cell, for shorter time delays we used a 20 Hz repetition rate, but in this case with 2000 pump–probe cycles of single X-ray pulse exposures (see Section 3). For longer time delays, we used 5 Hz repetition rate with a 24-pulse exposure (see Section 3) and 107 exposures per image, so the total number of single 100 ps X-ray pulses per image was 2568. As for the standard data collection, for each time delay we collected and averaged 50 images at both repetition rates.

For 5 Hz data collection, the sample flow rate was set to 5.45 µl min^−1^ and the buffer flow rate to 10 µl min^−1^. For 20 Hz data collection, the sample flow rate was 20.57 µl min^−1^ and the buffer flow rate was 28 µl min^−1^. The sample core was measuring ∼270 µm for the 5 Hz and ∼300 µm for the 20 Hz repetition rates. The measurements were performed at approximately 12 mm from the sample injection point. To ensure that the X-ray beam probes the sample volume excited by the laser beam, the laser beam position was calculated and offset for each time delay by data collection software. During switching of the frequency for the data collection in the co-flow cell, the sample and the buffer flow rates must change accordingly. During such switching, small variations in the sample core diameter are possible and the probed sample volume can change slightly. To account for the possible change of the X-ray path length in the sample at different repetition rates, a common data point at 500 µs time delay was collected to ensure proper scaling of the data.

A summary of the data collection parameters for the standard and co-flow cells is shown in Table 1 ▸. The table presents the repetition rate of the experiment, time delays used in the experiment, details of the sample refreshment, number of 100 ps X-ray pulses per image and sample volume consumption per image. As described in Section 3, for each recorded image we used repeated pump–probe cycles with single-pulse X-ray exposures for 20 Hz data collection (short pump–probe time delays) and 24-pulse X-ray exposures for 5 Hz data collection (longer pump–probe time delays). At both repetition rates for each time delay we collected and averaged 50 images for both standard cell and co-flow cell.

## 4. Results and discussion

**Figure 4:**
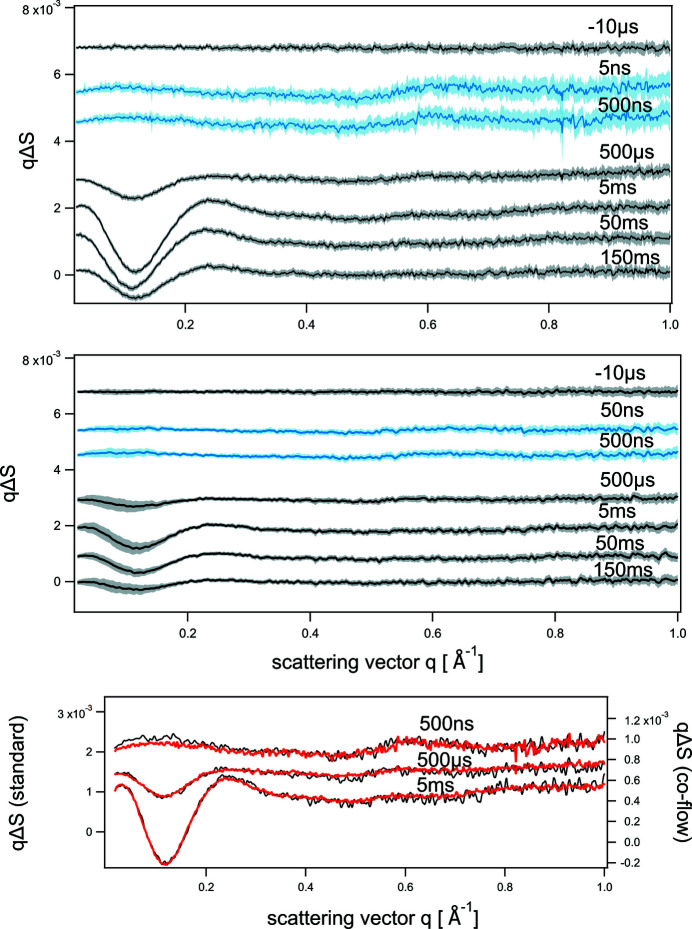
Difference signal *q*Δ*S*(*q*,*t*) as a function of scattering vector *q*, measured in the standard cell (top panel) and co-flow cell (middle panel). Black lines represent data collected at 5 Hz, blue lines those at 20 Hz. The bottom panel compares the shapes of difference scattering curves for the co-flow (black, right *y* axis) and standard (red, left *y* axis) data collections at selected time delays.

Fig. 4 ▸ shows difference signals *q*Δ*S*(*q*,*t*) as a function of scattering vector *q*. The difference signal was obtained by subtracting the laser-OFF reference scattering curve measured at −5 µs time delay from the scattering curves with the laser-ON positive time delays, containing the structural signal. The top panel shows difference data collected with the standard flow cell, and the middle panel shows difference data collected with the co-flow cell at different time delays. The data are presented on the same scale for direct comparison and are offset for clarity. The error band in the data represents root mean square deviation (RMSD) errors, obtained from averaging the data at each time delay. No median filters, sorting or outlier rejections were applied in the process of data averaging.

The signal-to-noise level for the data collected at 20 Hz (blue lines) at high scattering vectors (*q* > 0.4 Å^−1^) is smaller in the standard flow cell data than in other difference scattering curves. At 20 Hz, we performed measurements which demand single 100 ps X-ray pulse exposures. With the standard cell, these measurements take a long time because the sample has to be replenished multiple times in the capillary cell between the cell scans. Due to beam time limitations, the total number of 100 ps X-ray pulses per image that we used for the standard cell at 20 Hz was by a factor of ≥2 smaller compared with other data collection modes (see Table 1 ▸). In particular, with the standard flow cell at 20 Hz we used 1070 single 100 ps X-ray pulses per image (see Section 3.1), compared with 2000 single 100 ps X-ray pulses per image for the co-flow cell (see Section 3.2; for other details see Table 1 ▸).

At low scattering vectors (*q* < 0.3 Å^−1^) for the co-flow cell the RMSD errors of the averaged difference scattering curves are larger than in the standard cell measurements (Fig. 4 ▸). The signal-to-noise at lower scattering vectors is on average about four times larger for the standard cell than for the co-flow cell. We calculated the mean of the RMSD errors for averaged difference scattering curves for the 0.03 Å^−1^< *q* < 0.3 Å^−1^ region and determined that in this *q* region RMSD errors for all co-flow measurements exceed the RMSD errors in standard measurements by about two times. This is higher than expected, based on the differences in the sample volume in the two setups. We attribute this additional noise in the co-flow cell measurements to instabilities in the delivery of solutions caused by the syringe pump, which we used in the measurements. Despite this fact, the averaged scattering difference curves collected with both sample cells agree very well. The bottom panel of Fig. 4 ▸ compares difference scattering curves for 500 ns, 500 µs and 5 ms time delays. The curves for different time delays are offset for clarity. To emphasize the agreement between difference scattering curves from standard cell and co-flow cell measurements, the curves are presented using different absolute scales, shown on the left and right *y* axes. The scale on the left *y* axis corresponds to difference scattering curves measured in the standard cell axis. The scale on the right *y* axis corresponds to difference scattering curves measured in the co-flow cell. We observe excellent agreement in the pairs of difference scattering curves collected in the standard and co-flow configurations at the same time delays.

In Fig. 4 ▸, normalized time-resolved difference scattering signals for the co-flow cell are two times smaller than those for the standard cell. Such a difference is the result of the data normalization procedure. A standard procedure for the data normalization in TRXSS is normalization using the isosbestic region of the solvent *q* range of 1.4–1.6 Å^−1^. In the co-flow cell, all molecules of the buffer solvent, from the sample solution and from the sheath buffer, contribute to such signal. Therefore, normalized in this way, the structural signal appears proportionally smaller. In general, this is not a problem for the further data analysis. The scattering fingerprints of the intermediates are obtained by kinetic modelling and global analysis of the time-resolved scattering data (Cho *et al.*, 2016 ▸). A static scattering pattern of the protein ground state should be measured during the time-resolved (standard or co-flow) experiment. Then absolute scattering curves of the intermediates are acquired in the regular manner by adding the protein ground state scattering pattern and difference scattering pattern of the intermediate scaled for 100% photoexcitation (Cho *et al.*, 2016 ▸). Subsequently, the protein shape is modelled by using regular methods of static SAXS, or molecular dynamics simulations methods.

**Figure 5:**
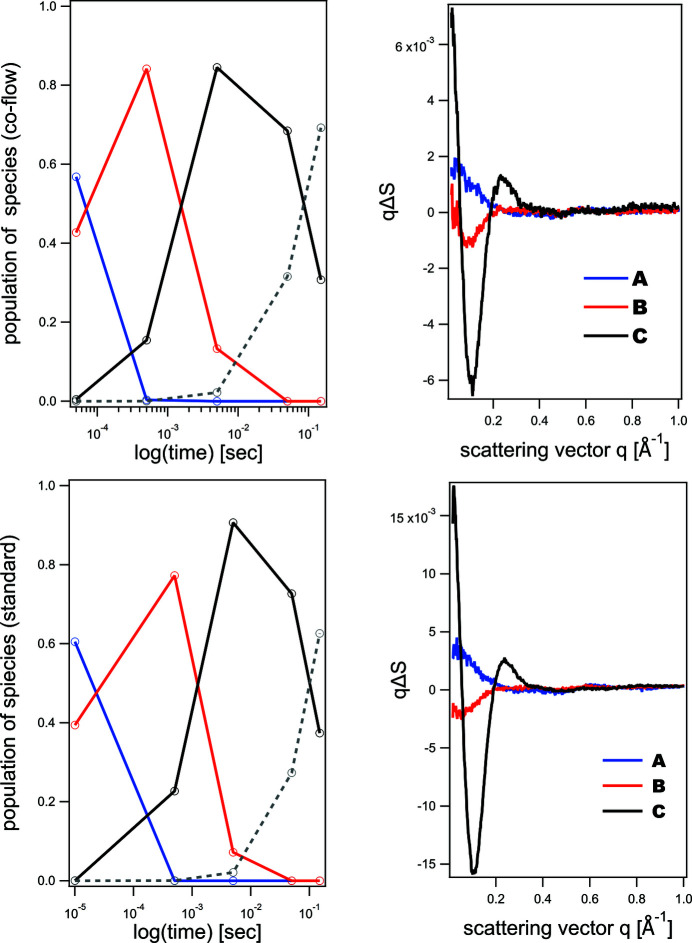
Right panels show difference scattering patterns as a function of scattering vector for PYP reaction intermediates *A* (blue), *B* (red), *C* (black): co-flow cell (top, right), standard cell (bottom, right). Time courses for the intermediate populations are shown in the left panels: co-flow cell (top, left), standard cell (bottom, left). The population time course of the ground state *D* is shown by the dashed lines. The difference scattering curve of the ground state *D* is equal to zero and is not shown.

Time-resolved scattering data were collected at only seven time delays, since detailed structural analysis of the PYP intermediate states was not the goal. However, we still performed global kinetic modelling of the available data for comparison purposes. For our analysis, we limited the scattering data to the *q* range 0.02–1.0 Å^−1^, the portion of the scattering curve where scattering is determined by structural changes of protein and protein–solvent interactions. For data analysis we used the *ReactLab Kinetics* program which is capable of modelling kinetic reactions of proteins of first and second order and is available commercially (https://jplusconsulting.com/products/reactlab-kinetics/). The result of the SVD analysis for both data collections indicated the presence of three intermediates. We modelled the data by following (Cho *et al.*, 2016 ▸) and applying a serial kinetic model *A* → *B* → *C* → *D* where *A*, *B* and *C* are intermediate species in the course of the reaction and *D* is the final ground PYP state. The population courses and difference scattering patterns of the reaction intermediates obtained by the kinetic modelling are shown in Fig. 5 ▸ (in the bottom panels for the standard cell and in the top panels for the co-flow cell). Both data protocols result in very similar scattering patterns of the intermediates, similar time constants and population courses. In particular, from global kinetic analysis, the time constants between states in the reaction *A* → *B* → *C* → *D* are comparable for the standard flow cell and the co-flow cell: 10 µs, 1.96 ms, 150 ms and 70 µs, 2.2 ms, 250 ms, respectively.

The differences in the time constants are likely attributed to sparse time-delay points and a minor difference is the time-delay sequence in the collected time domain (Table 1 ▸). Overall, the difference scattering patterns of the intermediates and their population’s time courses are in agreement with those observed in detailed time-resolved SAXS studies of the PYP photocycle (Cho *et al.*, 2016 ▸; Kim *et al.*, 2012*b* ▸). The final signalling state is observed with a relaxation time of 250 ms in the co-flow cell and 150 ms in the standard cell experiment. Those values are comparable with the relaxation time of the signalling state of 280 ms in the work of Cho *et al.* (2016 ▸), where a sequential model was also used to describe the PYP dynamics.

The most important comparison of the two devices is sample consumption. For the data collection described above, 57 ml of sample was used with the standard flow cell (7 ml for 5 Hz and 50 ml for 20 Hz data collection) and 9.5 ml was used for the co-flow cell (660 µl for 5 Hz and 8.8 ml for 20 Hz data collection). A comparison of the calculated sample consumption in a typical time-resolved pump–probe solution scattering experiment for capillaries of different sizes and for the co-flow device is presented in Table 2 ▸.

We show the calculated sample consumption for the standard cell capillary experiment in orthogonal laser beam/X-ray beam geometry for measurements conducted at 0.15 mm from the top of the capillary cell and compare it with the sample consumption of the co-flow cell. Both are for 20 Hz data collection used for short time delays and more demanding in terms of sample consumption (see Table 1 ▸). Owing to concerns about replenishing a liquid in the proximity of the capillary wall for the standard cell, we adopted the methodology of scanning the capillary through the series of pump–probe cycles, and then replenishing the sample (see also Section 3.1). To ensure maximum efficiency of the sample replenishing and to mitigate scattering structural difference errors due to the sample velocity profile at the proximity of the capillary wall, the pumped sample volume is typically two times larger than the calculated volume of the capillary cell device. In Table 2 ▸, column 5, we show the sample volumes per image in the standard cell and co-flow cell configurations. In column 6, we show the ratio of the sample volume per image in the standard cell and co-flow cell configurations. For the calculations in column 6, the co-flow sample volume was adjusted to account for X-ray sample path differences in the capillaries and in the co-flow cell, and to achieve identical total X-ray sample path per image in both devices. The experimental ratio values are substantially larger than the theoretical gains due to the adopted data collection protocol as described above.

## 5. Conclusions

We present a novel design of a sheath co-flow cell for minimizing sample consumption in time-resolved pump–probe X-ray solution scattering measurements. This design makes it feasible to study protein structural dynamics for irreversible reactions. By using photoactive yellow protein, we demonstrated that standard data collection at BioCARS 14-ID beamline and data collected with the sheath co-flow cell give similar difference scattering patterns for reaction intermediates and similar time constants from the kinetic analysis. We show that the co-flow cell uses significantly less sample than the standard capillary cell employed in BioCARS time-resolved pump–probe X-ray solution scattering experiments. Identical sample X-ray paths and exposures in both configurations can result in data with similar signal-to-noise levels providing improved stability of the pumps that deliver sample solution and sheath solvent buffer. Further advances can be achieved by improving the focusing X-ray optics and decreasing the X-ray and the laser beam sizes, and by utilizing novel integrating detectors with improved X-ray sensitivity.

## 6. Data availability

Authors will make available images for time-resolved measurements with photoactive yellow protein, averaged difference scattering patterns and results of the kinetic modelling upon reasonable request.

## Figures and Tables

**Table:** 

Standard data collection					
Repetition rate (Hz)	Time delays	Sample refreshment method	Capillary scans per image	100 ps X-ray pulses per image	Sample volume per image (µl)
5	−10 µs, −5 µs, 5 µs, 500 µs, 5 ms, 50 ms, 150 ms	Capillary scan, then refresh 20 µl	1	2568	20
20	−5 µs, −10 µs, 50 ns, 500 ns, 500 µs	Capillary scan, then refresh 20 µl	10	1070	200

**Table:** 

Co-flow data collection				
Repetition rate (Hz)	Time delays	Sample refreshment method	100 ps X-ray pulses per image	Sample volume per image (µl)
5	−10 µs, −5 µs, 50 µs, 500 µs, 5 ms, 50 ms, 150 ms	Flow	2568	1.89
20	−5 µs, −10 µs, 5 ns, 500 ns, 500 µs	Flow	2000	35.3

**Table 2:** Calculated sample consumption in standard cell and co-flow cell data collection

	Capillary diameter (mm)	Laser beam geometry relative to X-ray beam	X-ray path (mm)	Volume per image at 20 Hz (µl)	Experimental *V*_st_/*V*_co_ per image, corrected
Standard scan	1	Orthogonal 0.15 mm depth	0.714	1800	21.5
Standard scan	0.5	Orthogonal 0.15 mm depth	0.45	400	7.6
Standard scan	0.3	Orthogonal 0.15 mm depth	0.3	140	4
Co-flow	0.3	Pseudo-collinear	0.3	35	1
